# Health Department in Hyde Park

**Published:** 1879-08

**Authors:** William H. D. Lewis

**Affiliations:** Village Physician


### ARTICLE XV.

### HEALTH DEPARTMENT IN HYDE PARK.

Many physicians whose practice extends within the limits of this suburb, are unaware of the existence of the health department. For the benefit of such practitioners, I beg leave to respectfully call attention to this matter through the columns of the JOURNAL AND EXAMINER. The Health Department was established in September, 1878. The executive officer is the Village Physician who is appointed by the Board of Trustees. The duties of the Village Physician, in so far as physicians and midwives are concerned, are defined in the following selections from Chapter XXI of the Ordinances of the Village of Hyde Park:

"SECTION 1. Every practicing physician in the village, who shall have a patient laboring under yellow fever, small-pox or other infectious or pestilential disease, shall forthwith make a report thereof in writing, to the Village Physician describing the locality of the house or place where the said patient may be.

"SEC. 2. No person shall put out, remove or allow to be put out or removed, from the premises or place occupied or owned by him, into any street, alley or other public place in said village, any person having the small-pox or any other pestilential disease; but each owner or occupant shall immediately report such case to the Village Physician or the President of the Board of Trustees.

"SEC. 10. Every physician, midwife or other person who may professionally assist or advise at any birth or death, shall make a return of such birth or death to the Health Department of this village, such as is now required by the State Board of Health to the County Clerk. Said returns of births shall be made to said Health Department within ten days after the date of such birth, and returns of deaths shall be made to said Health Department within twenty-four hours after such death may occur. It shall be the duty of said Village Physician to transmit the returns of births and deaths aforesaid to the County Clerk within the time now prescribed by law.

"SEC. 12. No remains of any human being, having died within the corporate limits of the village, or whose body may have been found within the limits of the same, shall be removed from this village for interment, or otherwise, nor shall said remains be interred within the corporate limits of the Village of Hyde Park until the certificate, as required in Section 10 of this Chapter, shall have been filed at the office of the Health Department and a permit first had and obtained for each removal and interment."

It may be well to state that the Village of Hyde Park extends from 39th street to the Calumet river, and from State street to the lake, and includes the localities known as Oakland, Kenwood, Woodlawn, Egandale, Forrestville, Anthony, Park Side, Grand Crossing, South Chicago, Irondale, Holland Settlement, Wildwood and portions of Englewood, Riverdale, Colehour and Kensington.

Burial permits can be obtained upon the presentation of a death certificate from a physician, or the findings of a coroner's jury at Kenwood av, east 47th street, before 9 a. m. and after 7 p. m.; at the Village Hall at 10 a. m., or at 5001 State street (corner of 50th street) between 11 a. m. and 1 p. m., and 4 and 6 p. m., (Sunday, between 3 and 6 p. m).

Physicians are requested to report all cases of yellow, ship or typhus, typhoid, scarlet and spotted fevers, cholera, small-pox, chicken-pox, measles, whooping-cough and diphtheria. Blanks for this purpose will be furnished on application.

Cases of infectious diseases will be quarantined when necessary, and proper measures taken to prevent such diseases becoming epidemic.

The hearty co-operation of physicians in the requirements of the Health Department is earnestly solicited.

WILLIAM H. D. LEWIS,
*Village Physician.*

---

DR. WM. H. BYFORD having been invited to read a paper before the Obstetrical and Gynæcological Section of the British Medical Association, has sailed for Europe with that object in view.

